# Association of preoperative fasting blood glucose with 1-year Harris Hip Score after hip fracture in older adults: a multicenter retrospective cohort study

**DOI:** 10.3389/fendo.2026.1853284

**Published:** 2026-08-27

**Authors:** Huazhong Liu, Jiajun Liao, Ji Li, Guixin Zhang, He Tian, Huai Wu, Haoze Yang, Shiwei Yang, Yun Lu

**Affiliations:** 1 Shenzhen Pingle Orthopedic Hospital (Shenzhen Pingshan District Hospital of Traditional Chinese Medicine), Shenzhen, China; 2 Guangzhou University of Chinese Medicine, Guangzhou, China; 3 Northeastern University, Shengyang, China; 4 Peking University Shenzhen Hospital, Shenzhen, China; 5 Shenzhen Second People’s Hospital, Shenzhen, China

**Keywords:** functional recovery, Harris Hip Score, hip fracture surgery, multicenter study, preoperative fasting blood glucose

## Abstract

**Background:**

The impact of preoperative fasting blood glucose (FBG) on functional recovery after hip fracture remains uncertain.

**Methods:**

We conducted a multicenter retrospective cohort study of older adults undergoing hip fracture surgery at three hospitals in Shenzhen, China, between January 2021 and May 2023. De-identified electronic health record data were pooled across centers. The primary outcome was 1-year functional recovery, assessed using the Harris Hip Score (HHS) and categorized as satisfactory (HHS ≥70) or unsatisfactory (HHS <70). Associations between FBG and recovery were evaluated using logistic regression in unadjusted, demographic-adjusted, and fully adjusted models including 28 prespecified clinical and perioperative covariates. Sensitivity analysis additionally adjusted for study center. FBG was analyzed continuously and categorically using World Health Organization cutoffs and tertiles. Generalized additive models assessed dose–response patterns, and subgroup analyses explored effect modification.

**Results:**

Among 615 patients, higher preoperative FBG was associated with poorer 1-year functional recovery. In the fully adjusted model, each 1 mmol/L increase in FBG was associated with a 12% reduction in the odds of achieving HHS ≥70 (OR, 0.88; 95% CI, 0.80–0.96; P = 0.0036). Compared with FBG <6.1 mmol/L, FBG ≥7.0 mmol/L was associated with lower odds of satisfactory recovery (OR, 0.60; 95% CI, 0.37–0.98; P = 0.0414). Tertile analyses were consistent (highest vs lowest tertile: OR, 0.58; 95% CI, 0.34–1.00; P = 0.0485), with a significant linear trend. After additional adjustment for study center, the continuous association remained significant (OR, 0.89; 95% CI, 0.81–0.97; P = 0.0111), whereas categorical associations were attenuated. Smoothing curves suggested an approximately linear relationship. Subgroup findings were generally consistent but should be considered exploratory because no correction for multiple comparisons was applied.

**Conclusions:**

Elevated preoperative FBG was independently associated with worse 1-year functional recovery after hip fracture surgery, particularly when modeled continuously. FBG may aid risk stratification and perioperative planning, but threshold-based findings require further validation. Prospective studies should determine whether perioperative glycemic management improves functional outcomes.

## 1 Introduction

Geriatric hip fractures are a major cause of morbidity and mortality among older adults, with disability rates up to 50% and one-year mortality of 20–30% (1). These injuries—most commonly femoral neck, intertrochanteric, subtrochanteric, and acetabular fractures—occur predominantly in osteoporotic populations after low-energy falls, leading to marked loss of mobility, reduced independence, and substantial declines in quality of life (2). With global population aging, the incidence of hip fractures continues to rise and is projected to reach approximately 6 million cases worldwide by 2050, posing a significant burden on healthcare systems (3).

Early surgical intervention is the standard of care for geriatric hip fractures (4). Nevertheless, postoperative functional recovery varies widely and is influenced by multiple factors, including demographic characteristics (age, sex, and obesity) and comorbid conditions that affect microcirculation, such as hypertension, cardiovascular disease, and diabetes (5). Accurate identification of risk factors associated with postoperative Harris Hip Score (HHS) is essential for prognostication and individualized perioperative management.

Recent investigations have linked glycemic status to postoperative functional recovery. Hyperglycemia may impair fracture healing through several mechanisms (6–8). First, elevated glucose increases oxidative stress and promotes the formation of advanced glycation end-products in bone collagen, leading to osteoblast apoptosis, deterioration of bone microarchitecture, and disruption of the balance between bone formation and resorption. Second, hyperglycemia compromises host defenses, exacerbates local inflammation, and may heighten the risk of postoperative complications. Finally, endothelial dysfunction and microvascular injury reduce perfusion at the fracture site, slowing healing and limiting hip mobility.

Evidence regarding the impact of preoperative glycemia on hip-fracture outcomes remains mixed. Some studies support an association between higher glucose and worse prognosis: Shohat et al. (9) identified preoperative glucose control as a key prognostic factor; other investigations reported that inadequate perioperative fasting glucose management adversely affects outcomes (10). Tarabichi et al. (11) recommended maintaining perioperative hemoglobin A1c below 7.45%, and hyperglycemia has been reported as an independent 30-day surgical site infection risk factor in non-diabetic orthopedic trauma patients (12). Conversely, several studies found no significant association between preoperative glucose control and adverse postoperative events (13), and a prospective study reported no relationship between hyperglycemia and six-month mortality or functional outcomes in geriatric hip-fracture patients (14).

To address these inconsistencies, we conducted a multicenter cohort study integrating data from Shenzhen Second People’s Hospital, Peking University Shenzhen Hospital, and Shenzhen Pingle Orthopedic Hospital to evaluate the association between preoperative fasting blood glucose (FBG) and postoperative functional recovery, assessed by the 1-year HHS, in older adults with hip fracture.

## 2 Materials and methods

### 2.1 Study design and data sources

This multicenter retrospective cohort study was led by Shenzhen Second People’s Hospital in collaboration with Peking University Shenzhen Hospital and Shenzhen Pingle Orthopedic Hospital. We identified patients who underwent surgery for hip fracture between January 2021 and May 2023 at these three institutions. De-identified clinical data were extracted from each hospital’s electronic health record (EHR) system and merged for analysis. Variables included demographic characteristics, relevant medical histories, perioperative and surgical details, laboratory measurements, and 1-year follow-up documentation, including Harris Hip Score (HHS) assessments. All data handling complied with institutional data-protection policies.

Clinical Trial Registration: This study is a retrospective cohort study and does not involve prospective interventions. Therefore, a clinical trial registration number is not applicable.

The study protocol was approved by the Institutional Review Board/Ethics Committee of Shenzhen Second People’s Hospital (Approval No. [2025-161-01PJ]), Peking University Shenzhen Hospital (Approval No. [2025] (097)), and Shenzhen Pingle Orthopedic Hospital (Shenzhen Pingshan District Hospital of Traditional Chinese Medicine) (Approval No. [KY-2026-019]). In accordance with national regulations for retrospective research and given the minimal-risk design, the requirement for written informed consent was waived by the participating institutions. All procedures adhered to the principles of the Declaration of Helsinki and relevant ethical standards.

### 2.2 Inclusion and exclusion criteria

We included consecutive patients aged ≥60 years who underwent surgery for hip fracture at the three participating hospitals between January 2021 and May 2023. Hip fracture was defined as a femoral neck or intertrochanteric fracture confirmed by radiography or computed tomography. Patients from all centers were pooled; study center was not used as an eligibility criterion.

Exclusion criteria were: (1) old fractures, defined as >3 weeks between injury and hospital admission; (2) multiple or open fractures; (3) pathological or periprosthetic fractures; (4) refracture within 1 year after surgery; and (5) incomplete core data. Core data were prespecified as the 1-year Harris Hip Score (HHS) and preoperative fasting blood glucose (FBG).

**Figure 1:**
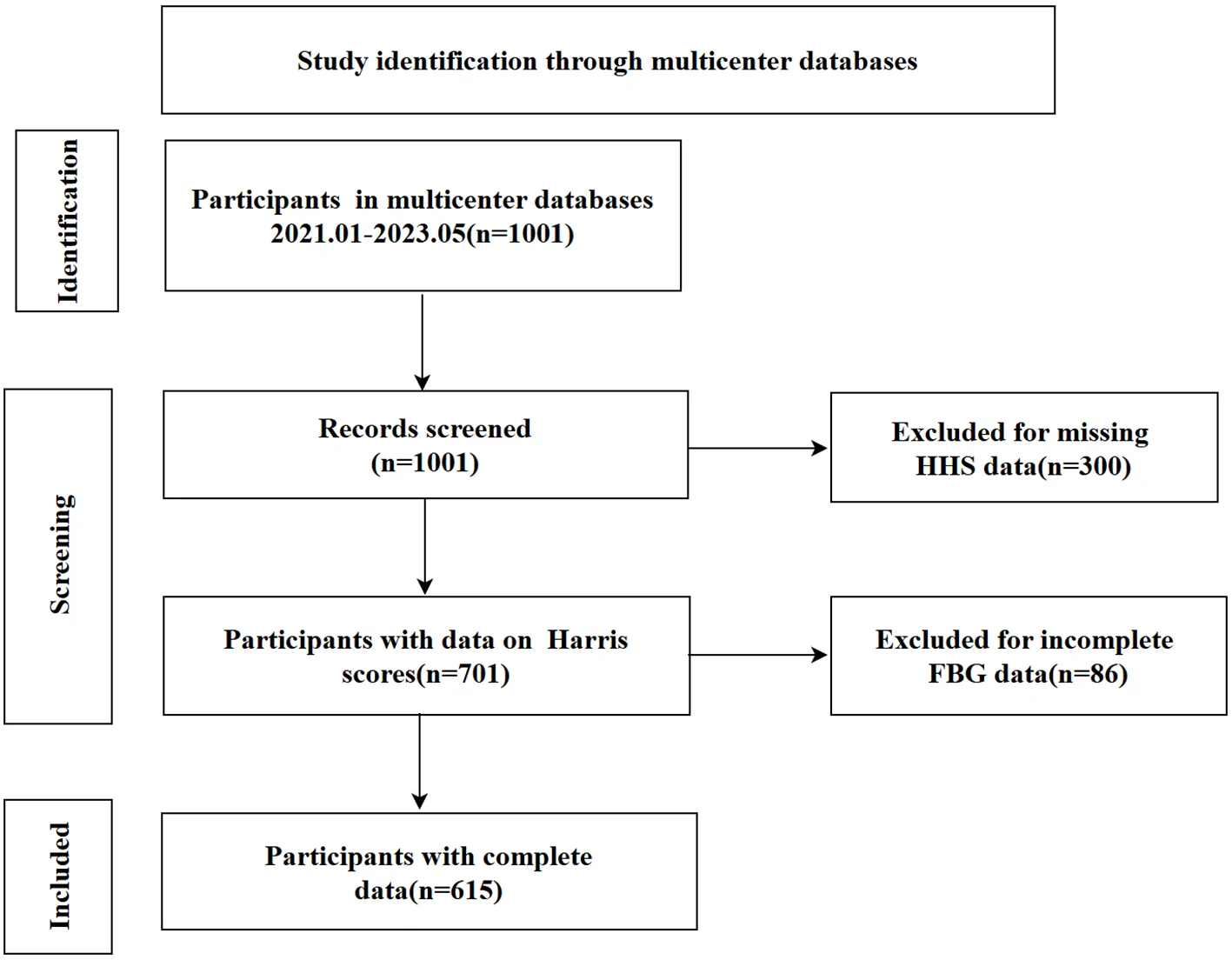
Flowchart of patient selection from the multicenter databases (January 2021–May 2023). Reasons for exclusion included missing 1-year Harris Hip Score (HHS) data (n=300) and missing preoperative fasting blood glucose (FBG) (n=86).

Of 1,001 patients screened, 386 were excluded because of missing core variables, including 300 patients without 1-year HHS data and 86 patients without preoperative FBG data, yielding a final analytical cohort of 615 patients. The selection process is shown in **Figure 1**.

### 2.3 Preoperative fasting blood glucose and postoperative Harris score

#### 2.3.1 Exposure: preoperative fasting blood glucose

Preoperative fasting blood glucose (FBG) was measured at the first clinical assessment on the day of admission or the following morning before surgery. Venous blood samples were obtained after an 8–10 h fast and analyzed in each hospital’s laboratory using the glucose oxidase method under uniform quality-control procedures. FBG was the primary exposure and was analyzed both continuously and categorically. According to World Health Organization (WHO) criteria (15), categories were defined as <6.1 mmol/L (normal), 6.1–6.9 mmol/L (impaired fasting glucose), and ≥7.0 mmol/L (suggestive of diabetes). To examine dose–response patterns, FBG was also grouped into tertiles based on its empirical distribution (Q1–Q3). FBG was chosen because it is routinely obtained during preoperative evaluation and provides a standardized fasting measurement of perioperative glycemic status. However, HbA1c was not routinely available across the three participating centers and therefore could not be incorporated into the present analysis. Accordingly, a single preoperative FBG measurement should be interpreted as reflecting glycemic status at admission rather than as a definitive marker of chronic dysglycemia.

#### 2.3.2 Perioperative glycemic management

Perioperative glycemic management was individualized according to each patient’s glucose level, diabetes history, nutritional status, comorbidities, surgical urgency, and the clinical judgment of the treating physicians. The general perioperative target glucose range was 5.6–10.0 mmol/L (100–180 mg/dL), consistent with current recommendations for hospitalized and perioperative patients (16, 17). Patients with FBG <6.1 mmol/L generally received routine perioperative care unless they had a prior diagnosis of diabetes requiring treatment. Patients with FBG of 6.1–6.9 mmol/L underwent routine perioperative glucose monitoring, and insulin was not routinely administered solely on the basis of this FBG range. For patients with FBG of 7.0–9.9 mmol/L, perioperative capillary glucose monitoring was strengthened, and correctional short-acting or rapid-acting insulin was considered if perioperative glucose exceeded 10.0 mmol/L or if hyperglycemia persisted. For patients with FBG ≥10.0 mmol/L or persistent perioperative glucose ≥10.0 mmol/L, glucose-lowering therapy was initiated or intensified, mainly using correctional insulin; scheduled insulin therapy, including basal or basal-bolus regimens, was considered for patients with persistent hyperglycemia or those already receiving insulin before admission (16, 17). Specific insulin type, dose, route, and timing were determined individually according to clinical condition.

#### 2.3.3 Outcome: postoperative Harris Hip Score

The primary outcome was the Harris Hip Score (HHS) at 1 year after hip fracture surgery. HHS assessments were primarily conducted by experienced orthopedic surgeons during scheduled outpatient visits. When in-person follow-up was not feasible, standardized telephone interviews were used to complete the assessment. Approximately 80% of the 1-year HHS assessments were completed through in-person outpatient visits, whereas approximately 20% were completed through telephone interviews. Before discharge, patients and their family caregivers received standardized instructions regarding the HHS-related functional assessment items and follow-up procedures. Family caregivers also completed two supervised practice assessments under the guidance of an experienced orthopedic assessor and demonstrated satisfactory understanding of the assessment procedures before follow-up. Telephone interviews were conducted by trained study personnel using the same HHS assessment framework as that used during in-person follow-up. These standardized pre-discharge procedures were implemented to reduce potential variability between telephone and face-to-face assessments, although a formal paired assessment of inter-method agreement was not performed. The HHS is a widely accepted instrument for hip function, comprising four domains—pain (44 points), function (47 points), deformity (4 points), and range of motion (5 points)—for a total score of 100 points. According to conventional HHS categories, scores are classified as excellent (90–100), good (80–89), fair (70–79), and poor (<70) (18). In the present study, we dichotomized the 1-year HHS at 70 points: patients with HHS ≥70 were classified as having satisfactory recovery, corresponding to at least a fair functional outcome and including the fair, good, and excellent categories; patients with HHS <70 were classified as having unsatisfactory recovery, corresponding to poor functional outcome. This threshold was used to distinguish patients who achieved at least fair hip function at 1 year from those with poor recovery.

We selected the HHS because it integrates pain and functional status, reflects postoperative quality of life, demonstrates good reliability and validity, and enables comparison with international studies (19). The 1-year time point was chosen because most patients have completed early rehabilitation and achieved substantial recovery by this stage, providing a robust indicator of longer-term functional outcomes and the influence of perioperative factors (20).

### 2.4 Covariates

To estimate the independent association between preoperative fasting blood glucose (FBG) and 1-year functional outcome, we adjusted for prespecified covariates selected from prior literature and clinical relevance. These included: Demographics and lifestyle: age, sex, body mass index (BMI), smoking status, and alcohol consumption. Comorbidities and medical history: hypertension, and prior stroke. Clinical and biochemical parameters: D-dimer, systolic and diastolic blood pressure, serum calcium, serum creatinine, urea nitrogen, uric acid, total cholesterol, triglycerides, N-terminal pro-B-type natriuretic peptide (NT-proBNP), Thrombocyte and white blood cell count. Hematologic indices: neutrophile granulocyte, hemoglobin. Perioperative and postoperative factors: preoperative transfusion, intraoperative transfusion, intraoperative blood loss, postoperative transfusion, postoperative deep vein thrombosis (DVT), fracture type (femoral neck or intertrochanteric), type of operation.

These covariates were entered into the primary multivariable models to reduce confounding when estimating the association between preoperative FBG and Harris Hip Score (HHS).Perioperative insulin use was not included in the multivariable models because detailed individual-level insulin administration data, including insulin type, dose, route, timing, and duration, were not uniformly available across the three participating centers.

### 2.5 Statistical analysis

Continuous variables were summarized as mean ± standard deviation, and categorical variables as counts and percentages. Between‐group differences were assessed using the Kruskal–Wallis test for continuous variables and the χ² test for categorical variables. To evaluate potential selection bias related to missing data, we compared available baseline characteristics between patients included in the final analytical cohort and those excluded because of missing 1-year HHS or preoperative FBG data. For this comparison, only observed data were used. The primary analytical cohort required complete data for the two prespecified core variables, namely preoperative FBG and 1-year HHS, and patients missing either variable were excluded. Missing values in other covariates were addressed before multivariable analyses.

The primary analysis used multivariable logistic regression with binary 1-year HHS recovery status as the outcome, defined as satisfactory recovery (HHS ≥70; fair, good, or excellent function) versus unsatisfactory recovery (HHS <70; poor function). Fasting blood glucose (FBG) was analyzed both continuously (per 1 mmol/L increase) and categorically using prespecified World Health Organization cut points (<6.1, 6.1–6.9, ≥7.0 mmol/L) and tertiles based on the empirical distribution. Odds ratios (ORs) and 95% confidence intervals (CIs) were reported. Linear trend across FBG categories was evaluated.

Three models were fitted: Model 1, unadjusted; Model 2, adjusted for demographic factors (age, sex, body mass index, smoking status, and alcohol consumption); and Model 3, fully adjusted for 28 prespecified confounders encompassing hematologic indices, physiologic parameters, comorbidities, and perioperative characteristics. To account for potential center-level differences across the three participating hospitals, we performed an additional sensitivity analysis by adding study center as a categorical covariate to the fully adjusted model.

To visualize the dose–response pattern and assess potential nonlinearity, generalized additive models (GAMs) with smooth curve fitting were applied to depict the relationship between preoperative FBG and the probability of satisfactory postoperative recovery. The GAM analysis was used as an exploratory visualization of the exposure–response pattern, whereas the primary effect estimates and P values were derived from the multivariable logistic regression models. Subgroup analyses were considered exploratory and hypothesis-generating. For the age-stratified subgroup analysis, participants were categorized into three groups according to tertiles of the empirical age distribution: lower age tertile (60–71 years), middle age tertile (72–80 years), and upper age tertile (81–98 years). No formal correction for multiple comparisons was applied; therefore, subgroup findings were interpreted cautiously, with emphasis placed on the overall pattern and interaction tests rather than on isolated stratum-specific estimates.

All analyses were performed using statistical software packages R (http://www.R-project.org, R Foundation) and EmpowerStats (http://www.empowerstats.com, X&Y Solutions Inc., Boston, MA, USA). Odds ratios (ORs) and 95% confidence intervals (CIs) were calculated. A two-sided P value <0.05 was considered statistically significant.

## 3 Results

### 3.1 Baseline characteristics of the participants

To assess potential selection bias related to missing data, we compared the available baseline characteristics between the included cohort and excluded patients. As shown in **Supplementary Table 1**, the included and excluded patients were generally comparable in sex, BMI category, smoking status, alcohol consumption, diabetes, hypertension, stroke, fracture type, systolic and diastolic blood pressure, hemoglobin, and serum creatinine. However, significant differences were observed in age, type of surgery, study center, urea nitrogen, and NT-proBNP, suggesting that missingness may not have occurred completely at random. In addition, baseline characteristics of patients excluded because of missing 1-year HHS data and those excluded because of missing preoperative FBG data are summarized descriptively in **Supplementary Table 2**.

**Table 1** presents the baseline characteristics of the study cohort, overall and stratified by postoperative Harris Hip Score (HHS; ≥70 vs <70). A total of 615 elderly patients underwent hip fracture surgery, with a mean age of 76.81 ± 8.63 years; 390 (63.41%) were women and 225 (36.59%) were men. The majority of patients had a body mass index (BMI) within the normal range of 18.5–24 kg/m² (470, 76.42%), while 43 (7.00%) were underweight and 102 (16.58%) were overweight. Lifestyle risk factors were uncommon, with only 6.67% of patients reporting smoking and 2.93% reporting alcohol consumption.

**Table 1:** Baseline characteristics by 1-year Harris Hip Score after hip fracture surgery.

Characteristic				*P*-value	*P*-value*
	Overall (N = 615)	HHS <70 (N = 181)	HHS ≥70 (N = 434)		
Demographics					
Age, years	76.81 ± 8.63	80.85 ± 8.42	75.12 ± 8.16	<0.001	<0.001
Sex				0.683	–
Male	225(36.59%)	64 (35.36%)	161 (37.10%)		
Female	390(63.41%)	117 (64.64%)	273 (62.90%)		
BMI, kg/m²				0.859	–
<18.5	43(7.0%)	14 (7.73%)	29 (6.68%)		
≥18.5, <24	470(76.42%)	136 (75.14%)	334 (76.96%)		
≥24	102(16.58%)	31 (17.13%)	71 (16.36%)		
Smoke				0.031	–
No	574(93.33%)	175 (96.69%)	399 (91.94%)		
Yes	41(6.67%)	6 (3.31%)	35 (8.06%)		
Alcohol				0.083	–
No	597(97.07%)	179 (98.90%)	418 (96.31%)		
Yes	18(2.93%)	2 (1.10%)	16 (3.69%)		
Comorbidities					
Diabetes				0.122	–
No	451(73.33%)	125 (69.06%)	326 (75.12%)		
Yes	164(26.67%)	56 (30.94%)	108 (24.88%)		
Hypertension				<0.001	–
No	279(45.37%)	62 (34.25%)	217 (50.00%)		
Yes	336(54.63%)	119 (65.75%)	217 (50.00%)		
SBP, mmHg	141.34 ± 21.31	142.03 ± 23.74	141.05 ± 20.20	0.602	0.396
DBP, mmHg	80.46 ± 11.56	79.54 ± 13.08	80.85 ± 10.85	0.2	0.184
Stroke				<0.001	–
No	440(71.54%)	105 (58.01%)	335 (77.19%)		
Yes	175(28.46%)	76 (41.99%)	99 (22.81%)		
Operative-related Factors					
Preoperative blood transfusion				0.002	–
No	555(90.24%)	153 (84.53%)	402 (92.63%)		
Yes	60(9.76%)	28 (15.47%)	32 (7.37%)		
Intraoperative blood transfusion				0.126	–
No	578(93.98%)	166 (91.71%)	412 (94.93%)		
Yes	37(6.02%)	15 (8.29%)	22 (5.07%)		
Postoperative transfusion				0.741	–
No	444(72.20%)	129 (71.27%)	315 (72.58%)		
Yes	171(27.80%)	52 (28.73%)	119 (27.42%)		
Intraoperative blood loss, ml	187.54 ± 155.45	176.66 ± 132.36	192.11 ± 164.34	0.262	0.46
Postoperative DVT				0.642	–
No	566(92.03%)	168 (92.82%)	398 (91.71%)		
Yes	49(7.97%)	13 (7.18%)	36 (8.29%)		
Type of fracture				<0.001	–
Femoral neck fracture	372(60.49%)	83 (45.86%)	289 (66.59%)		
Intertrochanteric fracture	243(39.51%)	98 (54.14%)	145 (33.41%)		
Type of surgery				<0.001	–
Total Hip Arthroplasty	301(48.94%)	55 (30.39%)	246 (56.68%)		
Femoral Head Replacement	57(9.27%)	30 (16.57%)	27 (6.22%)		
Internal Fixation	257(41.79%)	96 (53.04%)	161 (37.10%)		
Type of hospitals				0.014	–
Shenzhen Second People’s Hospital	189(30.73%)	44 (24.31%)	145 (33.41%)		
Shenzhen Pingle Orthopedic Hospital	275(44.72%)	97 (53.59%)	178 (41.01%)		
Peking University Shenzhen Hospital	151(24.55%)	40 (22.10%)	111 (25.58%)		
Preoperative Laboratory Tests					
FBG, mmol/L				<0.001	–
<6.1	269(43.74%)	60 (33.15%)	209 (48.16%)		
>=6.1, <7.0	113(18.37%)	31 (17.13%)	82 (18.89%)		
>=7.0	233(37.89%)	90 (49.72%)	143 (32.95%)		
Hemoglobin, g/L	116.49 ± 17.38	110.68 ± 17.37	118.89 ± 17.14	<0.001	<0.001
WBC count, ×10^9^/L	8.70 ± 2.62	8.78 ± 2.43	8.66 ± 2.69	0.625	0.521
NEU, ×10^9^/L	6.78 ± 2.55	7.01 ± 2.36	6.69 ± 2.63	0.154	0.071
SCR, μmol/L	77.51 ± 55.67	88.39 ± 70.28	72.90 ± 46.85	0.002	<0.001
UN, mg/dL	6.71 ± 3.11	7.54 ± 4.24	6.37 ± 2.43	<0.001	0.002
UA, μmol/L	300.32 ± 97.46	315.20 ± 109.70	294.35 ± 90.43	0.015	0.043
TCH, mmol/L	4.49 ± 0.86	4.34 ± 0.80	4.56 ± 0.88	0.003	0.042
TG, mmol/L	1.17 ± 0.57	1.13 ± 0.51	1.18 ± 0.60	0.297	0.237
NT-PROBNP, pg/mL	509.19 ± 832.60	671.42 ± 1099.47	440.02 ± 665.91	0.001	<0.001
Ca, mmol/L	2.20 ± 0.13	2.19 ± 0.14	2.21 ± 0.12	0.115	0.187
D-dimer, μg/L	8.18 ± 8.57	8.19 ± 7.43	8.17 ± 9.06	0.978	0.339
PLT, ×10^9^/L	206.98 ± 74.46	208.61 ± 72.31	206.29 ± 75.44	0.725	0.654

Values are presented as mean ± SD or n (%). P-value*: the Kruskal–Wallis rank-sum test was used for continuous variables.

FBG, fasting blood glucose; DBP, diastolic blood pressure; SBP, systolic blood pressure; WBC, white blood cell; NEU, neutrophil count; SCR, serum creatinine; UN, urea nitrogen; UA, uric acid; TCH, total cholesterol; TG, triglycerides; NT-proBNP, N-terminal pro-B-type natriuretic peptide; Ca, calcium; PLT, platelet count; DVT, deep vein thrombosis.

Hypertension was the most prevalent comorbidity (336, 54.63%), followed by prior stroke (175, 28.46%) and diabetes (164, 26.67%). In terms of fracture type, femoral neck fractures were more common (372, 60.49%) than intertrochanteric fractures (243, 39.51%). With respect to surgical management, total hip arthroplasty was performed in 301 patients (48.94%), internal fixation in 257 (41.79%), and femoral head replacement in 57 (9.27%).

The distribution of preoperative fasting blood glucose (FBG) showed that 269 patients (43.74%) had levels <6.1 mmol/L, 113 (18.37%) were between 6.1 and 7.0 mmol/L, and 233 (37.89%) had levels ≥7.0 mmol/L. According to the routine perioperative glycemic management protocols used in the participating hospitals, the target perioperative glucose range was 5.6–10.0 mmol/L. Patients with FBG below 10.0 mmol/L generally underwent routine or enhanced glucose monitoring according to their FBG level, diabetes history, nutritional status, comorbidities, and surgical urgency, whereas correctional insulin therapy was mainly considered for patients with perioperative glucose ≥10.0 mmol/L or persistent hyperglycemia. Detailed individual-level data on insulin type, dose, route, timing, and duration were not consistently available in the multicenter database.

Laboratory results indicated mean values of hemoglobin 116.49 ± 17.38 g/L, white blood cell count 8.70 ± 2.62 × 10^9^/L, serum creatinine 77.51 ± 55.67 µmol/L, and calcium 2.20 ± 0.13 mmol/L. Perioperative characteristics revealed that 60 patients (9.76%) required preoperative transfusion, mean intraoperative blood loss was 187.54 ± 155.45 mL, and postoperative deep-vein thrombosis occurred in 49 (7.97%).

Patients were recruited from three hospitals: Shenzhen Pingle Orthopedic Hospital (275, 44.72%), Shenzhen Second People’s Hospital (189, 30.73%), and Peking University Shenzhen Hospital (151, 24.55%). At 1-year follow-up, 434 patients (70.57%) achieved satisfactory recovery (HHS ≥70), while 181 (29.43%) had unsatisfactory recovery (HHS <70). Regarding the mode of 1-year HHS assessment, approximately 80% of patients were evaluated during in-person outpatient visits, whereas approximately 20% were assessed through standardized telephone interviews.

### 3.2 Associations between fasting blood glucose and Harris Hip Score

We next investigated the relationship between preoperative FBG and postoperative functional recovery using multivariable logistic regression models (**Table 2**). Across all three progressively adjusted models, elevated FBG was consistently associated with poorer functional recovery. In the fully adjusted model (Model 3), each 1 mmol/L increase in FBG was linked to a 12% reduction in the odds of satisfactory recovery (HHS ≥70) (OR = 0.88, 95% CI: 0.80–0.96, *P* = 0.0036).

**Table 2:** Association between preoperative fasting blood glucose and postoperative Harris hip score after hip fracture surgery.

Characteristic	Model 1	*P*-value	Model 2	*P*-value	Model 3	*P*-value
FBG	0.85 (0.79,0.91)	<0.0001	0.87 (0.81,0.95)	0.0008	0.88 (0.80, 0.96)	0.0036
Clinical threshold						
<6.1	1(reference)		1(reference)		1(reference)	
≥6.1, <7.0	0.76 (0.46, 1.26)	0.2836	0.84 (0.49, 1.42)	0.5107	0.89 (0.49, 1.61)	0.7027
>=7.0	0.46 (0.31, 0.67)	<0.0001	0.57 (0.37, 0.86)	0.0074	0.6 (0.37, 0.98)	0.0414
*P* for trend	<0.0001		0.0071		0.0384	
Tertiles						
Q1	1(reference)		1(reference)		1(reference)	
Q2	0.8 (0.51, 1.25)	0.33	0.91 (0.56, 1.46)	0.6831	0.89 (0.52, 1.54)	0.6761
Q3	0.45 (0.30, 0.70)	0.0004	0.56 (0.35, 0.88)	0.0123	0.58 (0.34, 1.00)	0.0485
*P* for trend	0.0003		0.0099		0.0378	

Data in table: OR (95% CI) *P***-**value.

Model 1: Unadjusted.

Model 2: Adjusted for age, sex, BMI, smoking and alcohol consumption.

Model 3: Adjusted for age, sex, BMI, smoking and alcohol consumption, D-dimer, blood pressure (SBP, DBP), serum calcium, hypertension, preoperative transfusion, intraoperative transfusion, intraoperative blood loss, postoperative transfusion, neutrophile granulocyte, NT-proBNP, hemoglobin, postoperative DVT, stroke, serum creatinine, Total cholesterol, triglycerides, urea nitrogen, uric acid, thrombocyte, white blood cell count, fracture type and type of operation.

Stratified analysis by WHO diagnostic thresholds further demonstrated that patients with FBG ≥7.0 mmol/L had a 40% lower probability of satisfactory recovery compared with those in the normal FBG group (<6.1 mmol/L) (OR = 0.60, 95% CI: 0.37–0.98, *P* = 0.0414). Importantly, although only a single FBG measurement was available, insufficient for diagnosing diabetes, patients in this range nonetheless exhibited significantly worse functional outcomes. Patients in the impaired fasting glucose group (6.1–6.9 mmol/L) showed a nonsignificant trend toward poorer recovery (OR = 0.89, 95% CI: 0.49–1.61, *P* = 0.7027). A significant linear trend across categories (P for trend=0.0384) underscored a dose–response relationship.

Subgroup analysis by tertiles yielded consistent findings. Compared with patients in the lowest tertile (Q1), those in the highest tertile (Q3) had significantly lower odds of satisfactory recovery (OR = 0.58, 95% CI: 0.34–1.00, *P* = 0.0485), with a significant linear trend across tertiles (*P* for trend=0.0378). Taken together, the primary analyses suggest that elevated preoperative FBG was associated with poorer 1-year functional recovery, with consistent findings across continuous, clinical-threshold, and tertile-based analyses.

To further account for potential center-level differences, we performed an additional sensitivity analysis by adding study center as a categorical covariate to the fully adjusted model. As shown in **Supplementary Table 3**, the association between FBG as a continuous variable and satisfactory recovery remained statistically significant after adjustment for study center (OR = 0.89, 95% CI: 0.81–0.97, *P* = 0.0111). However, the categorical associations based on clinical thresholds and tertiles were attenuated and no longer reached statistical significance, although the direction of effect remained consistent.

Clinically, these findings suggest that preoperative FBG may be useful as a readily available marker for risk stratification. However, threshold-based findings, including FBG ≥7.0 mmol/L, should be interpreted cautiously because the categorical associations were attenuated after additional adjustment for study center. Although causality cannot be confirmed within this study, the consistency of the association highlights the importance of glycemic monitoring and sets the stage for future intervention trials.

### 3.3 Linear relationship between the blood glucose level and Harris score in postoperative hip fracture patients

To further characterize the shape of the association between FBG and functional recovery, a logistic generalized additive model (GAM) with smooth curve fitting was employed. This flexible approach was used to visualize the exposure–response pattern and to assess whether there was evidence of nonlinearity.

**Figure 2:**
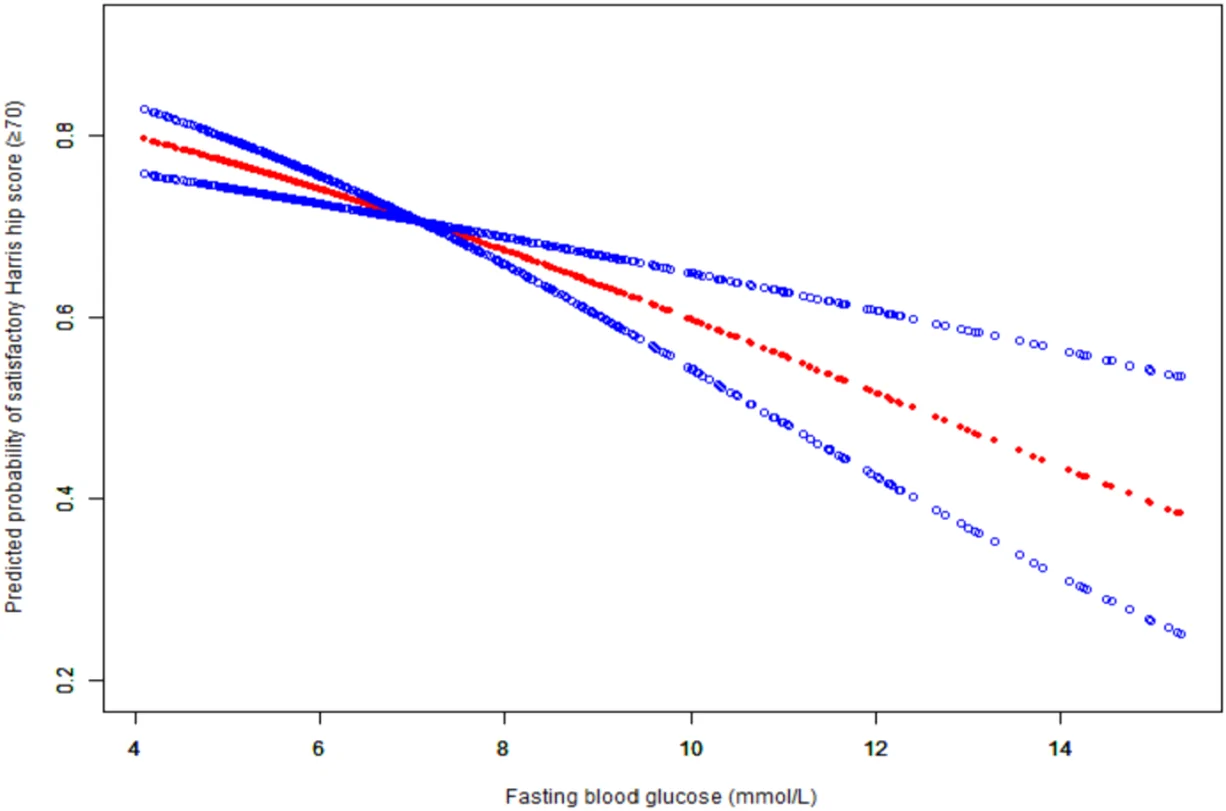
Logistic generalized additive model (GAM) showing the linear relationship between preoperative fasting blood glucose and postoperative functional outcome. The red line represents the fitted probability of achieving a satisfactory recovery (HHS≥70), while the blue lines indicate the 95% confidence interval.

The fitted smooth curve derived from the GAM, illustrated in **Figure 2**, was consistent with the primary logistic regression analysis and showed an approximately monotonic inverse association between preoperative FBG and the probability of achieving satisfactory recovery. As FBG levels increased, the probability of achieving HHS ≥70 decreased steadily, with no obvious threshold or inflection point.

Because the GAM was used primarily to visualize the exposure–response pattern and involved smooth curve fitting with a different modeling and testing framework, its P value is not directly comparable with the Wald P value from the primary multivariable logistic regression model. Therefore, the primary inference was based on the multivariable logistic regression results shown in **Table 2**. Overall, the GAM analysis supported the direction and approximate linearity of the association observed in the primary logistic regression models.

### 3.4 Subgroup analysis

**Figure 3:**
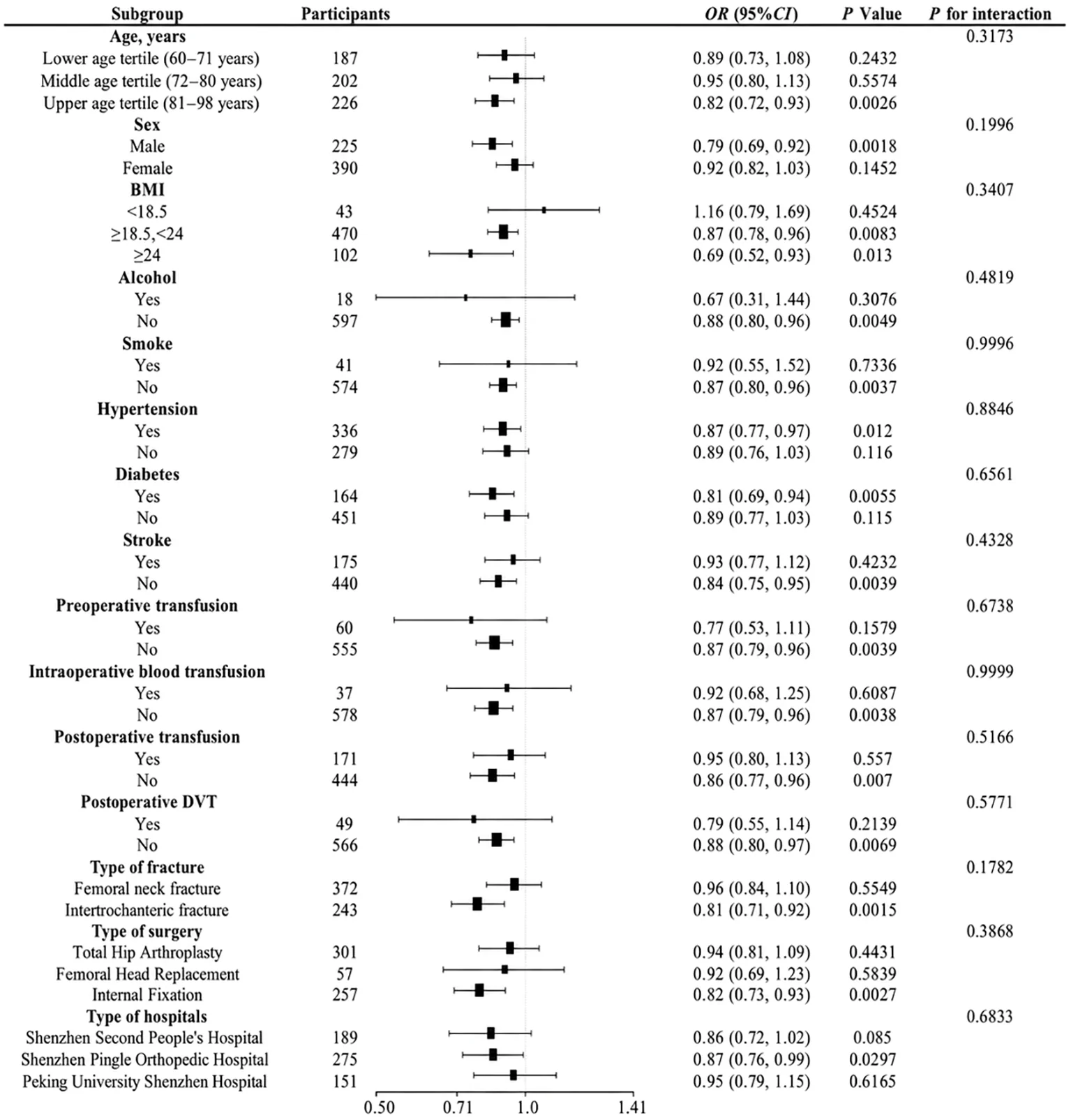
Subgroup analyses evaluating potential interactions between preoperative fasting blood glucose and covariates with respect to postoperative Harris hip score.

To explore potential effect modification, we conducted prespecified subgroup analyses according to patient characteristics, comorbidities, and perioperative factors (**Figure 3**). These analyses were exploratory and hypothesis-generating, and no formal correction for multiple comparisons was applied. Therefore, subgroup findings were interpreted cautiously, with emphasis placed on the overall direction of associations and interaction tests.

Overall, the inverse association between higher preoperative FBG and satisfactory recovery was directionally consistent across most subgroups. Numerically more pronounced associations were observed in the upper age tertile (81–98 years), males, participants with BMI 18.5–24 kg/m² or ≥24 kg/m², patients with hypertension or diabetes, those with intertrochanteric fractures, and patients treated with internal fixation. However, interaction tests did not provide strong evidence of statistically significant effect modification across these strata. Therefore, these subgroup findings should be interpreted as exploratory observations rather than confirmatory evidence of subgroup-specific effects.

## 4 Discussion

This study found that higher preoperative fasting blood glucose (FBG) was associated with poorer 1-year functional recovery in older adults after hip fracture surgery. In the primary fully adjusted model, higher FBG, analyzed as a continuous variable, was significantly associated with lower odds of satisfactory recovery. Analyses based on clinical thresholds and tertiles showed associations in the same direction. In the additional sensitivity analysis adjusting for study center, the continuous association remained statistically significant, whereas the threshold-based and tertile-based associations were attenuated. These findings suggest that the continuous relationship between higher FBG and poorer recovery may be more robust than specific threshold-based estimates. This attenuation may partly reflect center-level differences in patient composition, surgical decision-making, perioperative care pathways, rehabilitation practices, and follow-up patterns across the three hospitals.

Exploratory subgroup analyses showed that the inverse association between higher preoperative FBG and functional recovery was directionally consistent across most strata. However, interaction tests did not provide strong evidence of effect modification, and no formal correction for multiple comparisons was applied. These findings should therefore be interpreted cautiously and considered hypothesis-generating. Notably, even among individuals without a prior diagnosis of diabetes, FBG ≥7.0 mmol/L was associated with significantly poorer functional recovery, suggesting that elevated FBG may also be relevant in patients without known diabetes.

These findings expand upon previous research and underscore the importance of individualized perioperative glycemic management. Our results are consistent with substantial prior evidence emphasizing the role of glycemic control in surgical outcomes following fracture (9, 21, 22). The inverse association observed in our study is consistent with the findings of Semel et al. (23) and Gialanella et al. (24). both of whom identified abnormal glycemic status as a determinant of functional recovery. Prior studies have shown that diabetic patients not only recover more slowly and require longer hospital stays but also have a lower likelihood of direct discharge to home. Furthermore, evidence regarding glycemic thresholds supports our findings. Richards et al. (12) reported that two or more glucose measurements >11.1 mmol/L in non-diabetic trauma patients independently increased the risk of 30-day surgical site infections (OR 3.2, 95% CI: 1.3–7.8). Other research recommends maintaining FBG ≤7.8 mmol/L to reduce complications in hip fracture patients (25).

Additional studies have confirmed the association between hyperglycemia and postoperative complications. In orthopedic trauma, hyperglycemia has been identified as an independent risk factor for surgical site infection, even without diabetes (26). Jamson et al., studying 1,565 patients undergoing primary knee arthroplasty, found that perioperative glucose >6.9 mmol/L conferred a fourfold increased risk of infection compared with <6.1 mmol/L (27). Stress-induced hyperglycemia exceeding 12.2 mmol/L was associated with a sevenfold higher infection risk (28). These findings are especially relevant in acute hip fractures, where trauma itself can trigger stress hyperglycemia.

These findings are also consistent with clinical guidelines from the American College of Surgeons and the Surgical Infection Society, which recommend maintaining perioperative glucose between 6.1 and 8.3 mmol/L for all patients to reduce the risk of surgical site infections (29).

Nevertheless, some studies have reported divergent results. Thörling et al. (13) in a single-center observational cohort, found no significant association between admission HbA1c and postoperative complications or mortality after hip fracture surgery. Similarly, a prospective cohort study of hip and knee arthroplasty patients reported no definitive link between preoperative glycemic control and outcomes (30). Moreover, several prospective studies using the Harris Hip Score failed to show a significant relationship between hyperglycemia and 6-month functional outcomes in elderly hip fracture patients (14). These discrepancies may reflect differences in glycemic markers, outcome definitions, and the timing of assessment. Differences in sample characteristics, surgical techniques, statistical adjustment, and perioperative management may also have contributed.

Because HbA1c was not available in this study, we could not determine whether elevated preoperative FBG reflected chronic dysglycemia, acute stress-induced hyperglycemia after trauma, or both. Accordingly, the observed association should be interpreted as a relationship between admission fasting glycemic status and postoperative functional recovery, rather than as evidence that chronic dysglycemia alone caused poorer outcomes. Several mechanisms may provide biological plausibility for this association.

### 4.1 Inflammation and oxidative stress

Hyperglycemia activates NF-κB signaling, elevates cytokines such as TNF-α, and disrupts bone metabolism (31, 32). Hyperglycemia also increases reactive oxygen species, which may contribute to mitochondrial dysfunction, impaired bone matrix, and neutrophil dysfunction (33, 34).These changes may increase the risk of infection.

### 4.2 Abnormal bone metabolism

Chronic dysglycemia may promote the accumulation and cross-linking of advanced glycation end products in collagen, thereby reducing bone strength and inhibiting mesenchymal stem cell differentiation (35). Signaling through the receptor for advanced glycation end products may further disrupt osteoblast function and induce apoptosis (36, 37), while insulin resistance impairs Wnt/β-catenin signaling, limiting osteoblast activity and mineralization (38, 39). Inflammatory and neuroendocrine responses associated with acute stress may also contribute to impaired recovery. These mechanisms are biologically plausible but were not directly evaluated in the present study.

### 4.3 Microvascular dysfunction and muscle impairment

Hyperglycemia may damage endothelial cells and reduce perfusion at the fracture site (40, 41). It may also impair leukocyte function (42), and compromise muscle perfusion and nutrient delivery (43). These processes contribute to muscle atrophy and reduced functional capacity, reflected in lower Harris scores.

Together, these mechanisms provide biological plausibility for the observed association between elevated preoperative FBG and poorer functional recovery. From a clinical perspective, preoperative FBG may serve as a readily available marker for identifying patients who require closer perioperative glucose monitoring and individualized rehabilitation planning. In the participating hospitals, perioperative glycemic management generally aimed to maintain glucose within 5.6–10.0 mmol/L, with correctional short-acting or rapid-acting insulin considered mainly for perioperative glucose ≥10.0 mmol/L or persistent hyperglycemia, according to each patient’s clinical condition (16, 17). However, because detailed insulin administration data and longitudinal perioperative glucose measurements were not uniformly available, we could not determine whether specific glycemic interventions or insulin use improved postoperative functional recovery. Future prospective studies should collect standardized perioperative glucose trajectories and insulin-treatment data to clarify whether active glycemic management modifies long-term functional outcomes after hip fracture surgery.

## 5 Strengths

This study possesses several notable strengths. First, the study included a relatively large cohort from three medical institutions. This multicenter design increased the statistical power and broadened the applicability of the findings. Second, potential confounding factors were rigorously controlled through multivariable regression, and the dose–response relationship between fasting blood glucose (FBG) and functional outcomes was clearly demonstrated using smooth curve modeling. Third, prespecified subgroup and interaction analyses provided exploratory insights into patient groups in whom the association between FBG and functional recovery appeared numerically more pronounced, while these findings were interpreted cautiously as hypothesis-generating. Finally, this study provides multicenter real-world evidence suggesting that elevated preoperative FBG is associated with poorer functional recovery even among patients without a prior diagnosis of diabetes, thereby expanding current knowledge and offering new perspectives for perioperative risk stratification.

## 6 Limitations

Despite the significant associations observed, several limitations should be acknowledged. First, 386 of 1,001 screened patients were excluded because of missing 1-year HHS or preoperative FBG data. We compared available baseline characteristics between the included and excluded patients. We also separately summarized the characteristics of patients excluded because of missing 1-year HHS data and those excluded because of missing preoperative FBG data. Although many characteristics were comparable, significant differences in age, type of surgery, study center, urea nitrogen, and NT-proBNP suggested that missingness may not have occurred completely at random. Therefore, the exclusion of patients without 1-year HHS or preoperative FBG data may have introduced residual selection bias and may limit the external validity of the findings.

Second, the 1-year HHS was assessed using both in-person visits and telephone interviews. Although most assessments were conducted in person and all telephone interviews followed a standardized HHS assessment framework, telephone-based assessment may not fully capture certain physical examination components, such as range of motion or deformity, as accurately as face-to-face evaluation. This may have introduced some variability in outcome measurement. To reduce potential inter-method variability, patients and their family caregivers received standardized pre-discharge instruction, and family caregivers completed two supervised practice assessments under the guidance of an experienced orthopedic assessor. Telephone interviews were conducted by trained study personnel using the same HHS assessment framework as that used during in-person follow-up. Nevertheless, because formal agreement between telephone and face-to-face assessments was not evaluated, residual measurement differences cannot be excluded. Third, glycemic status was assessed using a single preoperative FBG measurement. HbA1c was not routinely available across the three participating centers and therefore could not be included in the analysis. As a result, we could not distinguish chronic dysglycemia from acute stress-induced hyperglycemia related to trauma, pain, inflammation, or acute physiological stress. This limitation may have led to exposure misclassification, and the findings should be interpreted as an association between preoperative fasting glycemic status and 1-year functional recovery rather than as evidence of a causal effect of chronic hyperglycemia. Fourth, although we described the routine perioperative glycemic management principles and target glucose range used in the participating hospitals, detailed individual-level data on insulin administration, including insulin type, dose, route, timing, and duration, were not uniformly available across the three centers. Therefore, perioperative insulin use could not be reliably quantified or included as a covariate in the multivariable models. Intraoperative and postoperative glucose fluctuations were also not recorded in sufficient detail, limiting our ability to evaluate whether perioperative glycemic variability or insulin treatment modified the association between preoperative FBG and functional recovery. Residual confounding related to perioperative glycemic management therefore cannot be excluded. Fifth, although multiple known confounders were adjusted for, the influence of unmeasured factors cannot be excluded. Sixth, although we performed an additional sensitivity analysis adjusting for study center, residual center-level differences may still exist, including differences in patient referral patterns, surgical decision-making, perioperative care pathways, rehabilitation protocols, and follow-up practices. After study-center adjustment, the association between continuous FBG and functional recovery remained significant, whereas categorical threshold-based associations were attenuated. Therefore, threshold-based findings should be interpreted cautiously. Finally, as an observational study, these findings demonstrate associations but do not establish causality. Prospective interventional studies are needed to clarify whether targeted glycemic control directly improves postoperative functional outcomes.

## 7 Conclusion

In this multicenter cohort study, higher preoperative fasting blood glucose (FBG) was consistently associated with poorer 1-year functional recovery after hip fracture. FBG was inversely associated with 1-year functional recovery, particularly when analyzed as a continuous variable. In the primary fully adjusted model, clinical-threshold and tertile-based analyses also suggested poorer recovery among patients with higher FBG. However, after additional adjustment for study center, the categorical associations were attenuated, whereas the continuous association remained statistically significant, suggesting that the continuous relationship was more robust than any specific threshold-based estimate. Exploratory subgroup analyses suggested numerically more pronounced associations in several strata, including relatively older patients, males, and patients with intertrochanteric fractures; however, these findings should be interpreted cautiously because no formal correction for multiple comparisons was applied and interaction tests did not provide strong evidence of effect modification. Plausible mechanisms include amplified inflammation and oxidative stress, abnormalities in bone metabolism, and microvascular dysfunction.

These findings support incorporating preoperative FBG assessment into routine evaluation to improve risk stratification and guide perioperative optimization. Patients with elevated preoperative FBG may warrant targeted glycemic assessment and individualized rehabilitation planning. The observed threshold values should not be interpreted as definitive clinical cutoffs but rather as exploratory findings requiring prospective validation. Future prospective studies, including randomized or pragmatic trials, should collect standardized perioperative glucose trajectories and insulin-treatment data to determine whether targeted glycemic management improves functional recovery, readmissions, costs, and long-term outcomes after hip fracture surgery.

## Data Availability

The de-identified data supporting the conclusions of this article are available from the corresponding author upon reasonable request, subject to approval by the relevant participating institutions and applicable ethical and data protection requirements.
