# Supplementary material for: Association of preoperative fasting blood glucose with 1-year Harris Hip Score after hip fracture in older adults: a multicenter retrospective cohort study
Source: Front Endocrinol (Lausanne). 2026 Aug 27;17:1853284. doi: 10.3389/fendo.2026.1853284 (PMC13558032; doi:10.3389/fendo.2026.1853284)
Supplement: Supplementary file 1 [file Table1.docx]

| **Variable** | **Included cohort (n=615)** | **Excluded patients (n=386)** | ***P*-value** | ***P*-value*** |
| --- | --- | --- | --- | --- |
| **Demographics** |  |  |  |  |
| **Age,years** | 76.81 ± 8.63 | 78.70 ± 9.02 | <0.001 | <0.001 |
| **Sex** |  |  | 0.883 | - |
| Male | 225 (36.59%) | 143 (37.05%) |  |  |
| Female | 390 (63.41%) | 243 (62.95%) |  |  |
| **BMI,kg/m²** |  |  | 0.058 | - |
| <18.5 | 43 (13.27%) | 54 (20.00%) |  |  |
| ≥18.5, <24 | 179 (55.25%) | 146 (54.07%) |  |  |
| ≥24 | 102 (31.48%) | 70 (25.93%) |  |  |
| **Smoke** |  |  | 0.663 | - |
| No | 574 (93.33%) | 362 (94.03%) |  |  |
| Yes | 41 (6.67%) | 23 (5.97%) |  |  |
| **Alcohol** |  |  | 0.867 | - |
| No | 596 (97.07%) | 373 (96.88%) |  |  |
| Yes | 18 (2.93%) | 12 (3.12%) |  |  |
| **Comorbidities** |  |  |  |  |
| **Diabetes** |  |  | 0.404 | - |
| No | 451 (73.33%) | 273 (70.91%) |  |  |
| Yes | 164 (26.67%) | 112 (29.09%) |  |  |
| **Hypertension** |  |  | 0.769 | - |
| No | 279 (45.37%) | 171 (44.42%) |  |  |
| Yes | 336 (54.63%) | 214 (55.58%) |  |  |
| SBP,mmHg | 141.34 ± 21.29 | 139.23 ± 20.41 | 0.122 | 0.083 |
| DBP,mmHg | 80.46 ± 11.55 | 79.24 ± 10.60 | 0.094 | 0.133 |
| **Stroke** |  |  | 0.695 | - |
| No | 440 (71.54%) | 271 (70.39%) |  |  |
| Yes | 175 (28.46%) | 114 (29.61%) |  |  |
| **Operative-related Factors** |  |  |  |  |
| **Type of fracture** |  |  | 0.715 | - |
| Femoral neck fracture | 372 (60.49%) | 229 (59.33%) |  |  |
| Intertrochanteric fracture | 243 (39.51%) | 157 (40.67%) |  |  |
| **Type of surgery** |  |  | <0.001 | - |
| Total Hip Arthroplasty | 301 (48.94%) | 163 (42.89%) |  |  |
| Femoral Head Replacement | 57 (9.27%) | 71 (18.68%) |  |  |
| Internal Fixation | 257 (41.79%) | 146 (38.42%) |  |  |
| **Type of hospitals** |  |  | <0.001 | - |
| Shenzhen Second People's Hospital | 189 (30.73%) | 221 (57.25%) |  |  |
| Shenzhen Pingle Orthopedic Hospital | 275 (44.72%) | 96 (24.87%) |  |  |
| Peking University Shenzhen Hospital | 151 (24.55%) | 69 (17.88%) |  |  |
| **Preoperative Laboratory Tests** |  |  |  |  |
| **Hemoglobin,g/L** | 116.47 ± 17.63 | 114.23 ± 19.15 | 0.065 | 0.216 |
| **SCR,μmol/L** | 77.46 ± 55.18 | 80.03 ± 57.64 | 0.498 | 0.781 |
| **UN,mg/dL** | 6.72 ± 3.12 | 7.32 ± 4.35 | 0.014 | 0.183 |
| **NT-PROBNP,pg/mL** | 508.12 ± 920.95 | 759.82 ± 1542.48 | 0.005 | 0.149 |

**Supplementary Table S1.** Comparison of baseline characteristics between included and excluded patients.

Note: Values are presented as mean ± SD or n (%). Comparisons were performed between the included cohort and all excluded patients using available observed data. Missing values were not imputed, and percentages were calculated based on the number of patients with available data for each variable. P-value*: for continuous variables, the Kruskal–Wallis rank-sum test was used; for categorical variables with an expected count of <10, Fisher’s exact test was used. FBG and HHS classification were not compared because missing preoperative FBG and missing 1-year HHS were exclusion criteria.

| **Variable** | **Missing 1-year HHS**  **(n=300)** | **Missing FBG**  **(n=86)** |
| --- | --- | --- |
| **Demographics** |  |  |
| **Age,years** | 79.19 ± 8.79 | 77.00 ± 9.63 |
| **Sex** |  |  |
| Male | 114 (38.00%) | 29 (33.72%) |
| Female | 186 (62.00%) | 57 (66.28%) |
| **BMI,kg/m²** |  |  |
| <18.5 | 43 (21.83%) | 11 (15.07%) |
| ≥18.5, <24 | 111 (56.35%) | 35 (47.95%) |
| ≥24 | 43 (21.83%) | 27 (36.99%) |
| **Smoke** |  |  |
| No | 280 (93.65%) | 82 (95.35%) |
| Yes | 19 (6.35%) | 4 (4.65%) |
| **Alcohol** |  |  |
| No | 290 (96.99%) | 83 (96.51%) |
| Yes | 9 (3.01%) | 3 (3.49%) |
| **Comorbidities** |  |  |
| **Diabetes** |  |  |
| No | 204 (68.23%) | 69 (80.23%) |
| Yes | 95 (31.77%) | 17 (19.77%) |
| **Hypertension** |  |  |
| No | 134 (44.82%) | 37 (43.02%) |
| Yes | 165 (55.18%) | 49 (56.98%) |
| SBP,mmHg | 139.56 ± 20.78 | 138.08 ± 19.14 |
| DBP,mmHg | 79.39 ± 10.94 | 78.70 ± 9.38 |
| **Stroke** |  |  |
| No | 204 (68.23%) | 67 (77.91%) |
| Yes | 95 (31.77%) | 19 (22.09%) |
| **Operative-related Factors** |  |  |
| **Type of fracture** |  |  |
| Femoral neck fracture | 183 (61.00%) | 46 (53.49%) |
| Intertrochanteric fracture | 117 (39.00%) | 40 (46.51%) |
| **Type of surgery** |  |  |
| Total Hip Arthroplasty | 128 (43.54%) | 35 (40.70%) |
| Femoral Head Replacement | 62 (21.09%) | 9 (10.47%) |
| Internal Fixation | 104 (35.37%) | 42 (48.84%) |
| **Type of hospitals** |  |  |
| Shenzhen Second People's Hospital | 151 (50.33%) | 70 (81.40%) |
| Shenzhen Pingle Orthopedic Hospital | 89 (29.67%) | 7 (8.14%) |
| Peking University Shenzhen Hospital | 60 (20.00%) | 9 (10.47%) |
| **Preoperative Laboratory Tests** |  |  |
| **Hemoglobin,g/L** | 113.85 ± 19.47 | 115.85 ± 17.75 |
| **SCR,μmol/L** | 81.00 ± 55.69 | 75.53 ± 66.26 |
| **UN,mg/dL** | 7.37 ± 4.51 | 7.06 ± 3.47 |
| **NT-PROBNP,pg/mL** | 821.41 ± 1606.95 | 486.21 ± 1190.27 |

**Supplementary Table S2.** Baseline characteristics of patients excluded because of missing 1-year HHS or missing preoperative FBG data.

Note: Values are presented as mean ± SD or n (%). The missing 1-year HHS and missing FBG groups were summarized separately using available observed baseline data. Missing values were not imputed. Percentages were calculated based on the number of patients with available data for each variable. FBG and HHS classification were not included in this descriptive table because missing preoperative FBG and missing 1-year HHS were exclusion criteria. No P values are reported because this table is intended to describe the baseline characteristics of patients excluded for each missing-data reason rather than to perform formal between-group comparisons.

| **Characteristic** | **Model 1** | ***P*-value** | **Model 2** | ***P*-value** | **Model 3** | ***P*-value** |
| --- | --- | --- | --- | --- | --- | --- |
| FBG | 0.85  (0.79,0.91) | <0.0001 | 0.87  (0.81,0.95) | 0.0008 | 0.89  (0.81, 0.97) | 0.0111 |
| Clinical threshold |  | | | | | |
| <6.1 | 1(reference) | | 1(reference) | | 1(reference) | |
| ≥6.1, <7.0 | 0.76  (0.46, 1.26) | 0.2836 | 0.84  (0.49, 1.42) | 0.5107 | 0.91  (0.50, 1.65) | 0.7511 |
| >=7.0 | 0.46  (0.31, 0.67) | <0.0001 | 0.57  (0.37, 0.86) | 0.0074 | 0.65  (0.40, 1.07) | 0.0892 |
| *P* for trend | <0.0001 | | 0.0071 | | 0.0839 | |
| Tertiles |  | | | | | |
| Q1 | 1(reference) | | 1(reference) | | 1(reference) | |
| Q2 | 0.8  (0.51, 1.25) | 0.33 | 0.91  (0.56, 1.46) | 0.6831 | 0.92  (0.53, 1.60) | 0.7785 |
| Q3 | 0.45  (0.30, 0.70) | 0.0004 | 0.56  (0.35, 0.88) | 0.0123 | 0.64  (0.37, 1.10) | 0.1081 |
| *P* for trend | 0.0003 | | 0.0099 | | 0.0888 | |

**Supplementary Table S3.** Association between preoperative FBG and postoperative HHS after additional adjustment for study center.Data in table: OR (95% CI) *P***-**value.
**Model 1:** Unadjusted.
**Model 2:** Adjusted for age, sex, BMI, smoking and alcohol consumption.
**Model 3:** Adjusted for age, sex, BMI, smoking and alcohol consumption, D dimer, blood pressure (SBP, DBP), serum calcium, hypertension, preoperative transfusion, intraoperative transfusion, intraoperative blood loss, postoperative transfusion, neutrophile granulocyte, NT-proBNP, hemoglobin, postoperative DVT, stroke, serum creatinine, Total cholesterol, triglycerides, urea nitrogen, uric acid, Thrombocyte, white blood cell count, fracture type, type of operation, and Type of hospital.
